# The Role of PEDF in the Eye, Bone, and Nervous and Immune Systems

**DOI:** 10.3390/pharmaceutics17081064

**Published:** 2025-08-15

**Authors:** Krittikan Chanpaisaeng, Crispin R. Dass

**Affiliations:** 1 Department of Pharmacology and Physiology, Faculty of Pharmaceutical Sciences, Chulalongkorn University, Bangkok 10330, Thailand; krittikan.c@chula.ac.th; 2 Center of Excellence for Preclinical Toxicity and Efficacy Assessment of Medicines and Chemicals, Chulalongkorn University, Bangkok 10330, Thailand; 3 Curtin Medical School, Curtin University, Kent St., Bentley 6102, Australia; 4 Curtin Medical Research Institute, Curtin University, Kent St., Bentley 6102, Australia; 5 Faculty of Pharmacy, Silpakorn University, Nakhon Pathom 73000, Thailand

**Keywords:** PEDF, serpin, eye, bone, nervous system, immune system

## Abstract

This review highlights recent findings on the versatile inactive serpin protein, pigment epithelium-derived factor (PEDF) in the eye, bone, and nervous and immune systems. PEDF is highly conserved and found at the 17p13.3 locus in humans. PEDF initially discovered in the eye, also has critical roles in the bone including de novo bone regeneration. It is also involved in the nervous system, with roles in such widespread and increasing-in-prevalence conditions such as depression, orchestrating the immune system, coordinating immune cells, and warding off disease. This manuscript comprehensively reviews the protein, listing a majority of all the publicly available studies reported, to date, in these four separate body systems. It thus showcases PEDF’s versatility in the human body. It also highlights the applicability of PEDF peptides, shorter in length, and in some cases just as potent as the parent protein in these discussed ailments.

## 1. Introduction

The PEDF gene is first believed to have appeared in vertebrates [1]. The PEDF gene is well-conserved amongst vertebrates, ranging from 48.3 kb in *Xenopus tropicalis* to 2.9 kb in fugu, with humans coming in at 15.6 kb [1]. The human PEDF gene is found on chromosome 17 (loci 17p13.3), consists of 9 exons, and codes for a protein 418 amino acids long. PEDF, a serine protease inhibitor superfamily (SERPIN) member, has a reactive centre loop (RCL) [2]. Three β-sheets and ten α-helices make up its secondary structure [3]. Incidentally, *CRK*, *PAFAH1B1*, and *YWHAE* genes, also at chromosome 17p13.3 all have critical roles in migration of neurons [4], and the story is the same for PEDF which is known to have an effect on neuronal stem cell survival (reviewed in [5]). Could PEDF have broader mental links that remain to be identified? Time will tell, and further research is warranted.

**Figure 1:**
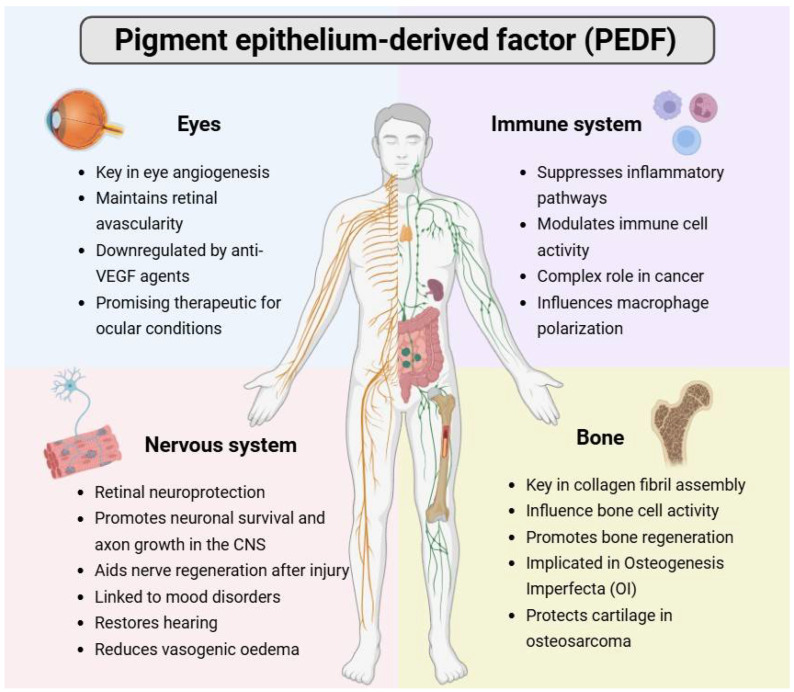
Function of PEDF in the eye, bone, and nervous and immune systems in the body.

Serpins include antichymotrypsin and antitrypsin [6]. PEDF is activated by chymotrypsin cleavage, though it lacks protease inhibitory activity [7]. While PEDF in the blood is largely thought to arise from liver and adipose tissue [8], the serpin has been discovered to be ubiquitously expressed in various mouse and human tissues, such as fat, blood, brain, and bone [9,10,11]. This discussion paper looks at the roles played by PEDF in the eye, bone, nervous, and immune systems (previewed in Figure 1).

## 2. PEDF and the Eye

Perhaps the bulk of research in angiogenesis, and a large portion in general, are based on studies of PEDF in the eye. However, this current paper does not deal with angiogenesis and PEDF directly, but indirectly in relation to the eye and to some extent the bone (discussed later on).

In PEDF KO mice, GM-CSF and IL-2 were elevated in retinas, correlating with increase in infiltration of macrophages, glia activation, pathologies linked to the vasculature, loss of photoreceptors, swelling of the inner plexiform layer, and consequent visual loss [12]. Cells belonging to the retinal pigment epithelium (RPE) secrete PEDF apicolaterally, where it deposits in the interphotoreceptor matrix, and maintains its avascularity [13]. In PEDF KO mice, several ocular surface and lacrimal gland (LG) disturbances were noted, including LG weight, sensitivity of the cornea, tear film, damage to the ocular surface, and acini size making up the LG [14].

In RPE cells grown under normoxic conditions, addition of anti-VEGF agents (bevacizumab, ranibizumab, and aflibercept) resulted in a downregulation of PEDF [15]. This was similar to the case when RPE cells were under hypoxia and could be due to the fact that PEDF is no longer required to control awry vascularisation in the eye. Incidentally, PEDF was initially identified as the most potent anti-angiogenic protein in the eye, specifically in a VEGF-induced corneal neovascularisation (CNV) model [16].

In the retina, PEDF protects retinal cells against oxidative stress [17], has an impact on the differentiation of photoreceptors (PHRs), and supports their survival [18], and prevents light-induced cell death [19]. Effects of PEDF on PHR survival and development were studied in neuronal retinal cell cultures, where PEDF, 17-mer and 44-mer PEDF-derived peptides protected PHRs from apoptosis, preserving mitochondrial function, promoting differentiation (polarisation of opsin), and stimulating axonal outgrowth [20].

AMD (age-related macular degeneration), found in the retinal macula, and known to be chronic and progressive, causes irreversible central vision loss in old people. It can cause irreversible blindness in this demographic, and apoptosis and inflammation are part of its pathogenesis. In a model of progressive, focal retinal degeneration, PEDF levels are lower than in WT mice retina and RPE [21]. When these mice were treated with PEDF, treated eyes exhibited less rate of progression or change in the focal retinal lesions, smaller and/or fewer photoreceptors, and degeneration of the RPE.

In cases of rhegmatogenous retinal detachment (RRD), in its neuroprotective role, PEDF protects the retina [22]. Recently, it was found that PEDF was elevated in patients suffering from RRD and endophthalmitis. Furthermore, in RRD patients, PEDF levels were higher if the tear was found below the retina, if there was detachment of the macula, and if RRD lasted longer [23]. These findings suggest that PEDF is upregulated as a result of the RRD condition, but no direct testing to confirm this has been performed, to date.

So, what treatments options are available for ocular conditions? Recently, topical administration of PEDF improved graft survival rate of corneal allografts in rats, reducing haemangiogenesis and infiltration of immune cells into the cornea [24]. In particular, numbers of type 17 T helper cells were increased but regulatory T cells were decreased. Promisingly, PEDF increased nerve reinnervation within grafts in these animals.

When intravitreal administration of various treatments was evaluated in an oxygen-induced retinopathy (OIR) model in rats, PEDF per se or combined with anti-VEGF drugs reduced neovascularization-related pathology and vessel damage, better than anti-VEGF drugs alone [25]. The combination of PEDF and VEGF antagonist was more potent in treating arterial tortuosity.

These findings demonstrate that PEDF maintains healthy retinal cells through antioxidant effect as well as regulates nerve reinnervation and vascularisation by working counteractively with VEGF. While endogenous PEDF is downregulated by anti-VEGF agents, administering exogenous PEDF together with VEGF antagonists showed promising benefits on preventing abnormal blood vessel growth.

## 3. PEDF and the Nervous System

There are numerous studies associating PEDF with the nervous system, and what happens when PEDF is underexpressed. For instance, PEDF receptor (PEDF-R) and laminin receptor (LR, also a receptor used by PEDF) are both crucial for survival of retinal neurons as through them, PEDF mediates pro-survival effects of PEDF on retinal ganglion cells (RGCs) [26]. A recent study showed that PEDF targets VEGFR-1/Flt-1, which is significant to PEDF-caused retinal neuroprotection [27]. Further studies are needed to confirm PEDF’s binding to VEGFR-1, whether it can bind to other VEGF receptors, and which conditions are amenable to exploit such binding for potentially therapeutic purposes.

PEDF is within adipose tissue-derived stem cell (ASC) secretome [28]. Secreted factors can have a positive impact on CNS cell populations. The factor was found to be one of the proteins found in the secretome to support post-natal neuronal survival, stimulate neurodifferentiation, and incite axonal growth in cell culture. PEDF serum levels correlate positively with CSF levels, which are increased in overweight patients and in type 2 diabetic patients, and levels increased with blood–brain barrier (BBB) dysfunction [29]. PEDF’s role in restoration of lesioned central nervous system is also shown where improved motor behavioural performance was seen with PEDF administration in mouse model of lower thoracic SC photothrombotic ischaemia [30].

Major depressive disorder (MDD) is a mental disease characterised by duration for over two weeks [31]. PEDF levels were significantly reduced in the plasma of initial-episode MDD patients, [32] as well as in the peripheral blood exosomes [33]. PEDF was also found to be decreased in the periphery and hippocampus of two depression animal models. As proof of involvement, hippocampal PEDF reduction resulted in depressive-like behaviours, synaptic impairments, and aberrant Wnt mechanisms, while elevated PEDF had opposite results. In support of this, another study using a murine model of CUMS revealed that restoring PEDF lessened behaviours associated with depression, such as decreased interest in sucrose and increased passivity in swimming tests [33].

Depression manifests itself as sadness, despair, lack of interest, and reduced cognition [34], placing significant burden on families, friends, and society on various levels. MDD patients display lower levels of PEDF in their circulation [35]. PEDF expression in the prefrontal cortex (PFC) was reduced in a murine model of chronic social defeat stress and rat model of chronic unpredictable mild stress. Conversely, in PEDF-overexpressing wildtype mice, the PFC had features that showed resistance to depression.

Several studies of PEDF and the CNS took place in the nervous system in the eye and are discussed here rather than above (eye section). For instance, PEDF can reduce, or even inhibit, apoptosis of retinal ganglionic cells (RGCs) occurring after optic nerve injury by elevating Bcl-2 protein levels or Bax protein reduction [36]. Both RGC number and morphology were better in the PEDF cohort compared to its untreated counterpart. Human RPE cells with overexpressed PEDF or pretreated with recombinant PEDF had increased glutathione levels post-H_2_O_2_ incubation [37]. PEDF-treated RGC reduced inflammatory reactions and degeneration in cells.

When neural stem cell (NSC) expressing PEDF were delivered to the retinal ganglion cell (RGC) post-optic nerve injury, it led to heightened RGC survival and enhanced axon regeneration in injured nerves [38]. PEDF secreted from Müller cells promotes RGC survival through STAT3 signalling [39]. Therefore, boosting Müller cell secretion may promote retinal ganglion cell (RGC) survival in neurodegenerative diseases involving the retina.

In our body, neuronal cells lost to trauma or injury are not replaced, as axons fail to regenerate, thus leading to a permanent functional deficit in functioning of the affected part of the CNS. In an earlier study [40,41], it was revealed that administration of PEDF promoted adult RGC neuroprotection and axon regeneration. Short-term treatments with PEDF decreases intracellular calcium in murine photoreceptors [42]. PEDF thus mediates lower calcium levels, thereby rescuing degenerating photoreceptor cells from death.

In a subsequent evaluation [43], it was demonstrated that PEDF is highly elevated in dorsal root ganglion neurons (DRGNs) from models entailing regenerating dorsal column trauma when compared to non-regenerating models for DC injury. PEDF is neuroprotective to adult DRGNs and permits outgrowth of neurites, whilst overexpression of PEDF after damage to DC in vivo promotes significant axon regeneration with boosted physiological function.

Status epilepticus is a common neurological emergency and is a prominent risk factor for epilepsy [44]. It may lead to neuronal death, plus boost serum ending up in the brain parenchyma (vasogenic oedema) causing severe complications [45]. A PEDF derivative (PEDF 335) was found to activate 67LR signalling, raising the possibility of it being used to control vasogenic oedema [46].

In rabbits undergoing surgery, the 44-mer peptide plus docosahexaenoic acid (DHA) is capable of stimulating corneal nerve regeneration and increasing sensitivity and tear secretion [47]. Blockade of adipose triglyceride lipase reduces such activity when full length PEDF and DHA are used [48]. In PEDF KO mice, elevated corneal injury and tears, and decreased corneal innervation and sensitivity were observed [49]. In PEDF-R KO mice retinas, the build-up of lysophosphatidyl choline-DHA and lysophosphatidyl ethanolamine-DHA suggests these lipids may contribute to the impaired photoreceptor function associated with PEDF deficiency [50].

Tissue perivascular resident macrophages (PVM/Ms) are needed for furnishing the endocochlear potential needed for hearing [51]. PVM/Ms regulate adherens- and tight-junction proteins in the endothelial barrier of the stria vascularis through secretion of PEDF. PEDF administered to the damaged ear restores intrastrial fluid–blood barrier integrity, thereby reducing hearing loss.

More recently, PEDF was conjugated to a prestin-targeting peptide 2 (PrTP2) which allowed the serpin to be targeted to prestin and accumulated around sensory outer hair cells (OHCs) for sustained release, thereby reducing OHC and spiral ganglion neuron (SGN) hearing loss [52]. Tests revealed that hearing loss was reverted with protection of these cells. In a sciatic nerve rat injury model, PEDF was found to increase both area and number of myelinated axons [53]. Upon exposure to PEDF, oxidative regulation, by way of glutathione peroxidase, superoxide dismutase, and catalase all went up.

In an ex vivo model of ocular ischaemia/hypoxia in rats, PEDF inhibited labyrinth angiogenesis and kept the capillary lumen patent [54]. It also reduced the number of apoptotic ganglion and inner nuclear layer cells. The neurotrophic-region-containing PEDF peptides decreased the number of apoptotic photoreceptors in retinal degeneration models in mice [55]. PEDF levels in the RPE of diseased mice decrease with age (P15–P25), while PEDF-R levels also decline in the photoreceptor inner segments. Conversely, neutralising PEDF via antibodies in RPE-CM increased retinal apoptosis [56].

Induced pluripotent stem (iPS)-RPE cells secrete more than ten-fold more levels of PEDF than mesenchymal stem cells (MSCs) and hundred-fold more than (neural stem cells) NSCs in vitro [57]. Transplanted iPS-RPE cells released a significantly high concentration of PEDF in vivo from shortly after transplantation (approximately hundred-fold compared to NSC- and four-hundred-fold higher compared to MSC-transplanted eyes at PODs 7 and 14) for more than three weeks. This was suggested to contribute to protection by RPE transplantation.

PEDF’s role and regulation in ocular cells are multifaceted. PEDF increases Bcl2 transcript levels, thereby halting apoptosis, in serum-starved retinal cells [58]. Induction of ER stress in Müller cells increases VEGF expression but decreased PEDF expression [59]. In RPE cells under normoxic conditions, VEGF inhibitors (bevacizumab, ranibizumab and aflibercept) downregulated PEDF [15]. In diabetic mice, hyperglycaemia reduced endogenous VEGF-B expression in corneal epithelium that has been regenerated, and exogenous VEGF-B led to recovery of corneal innervations and trophic functions [60]. VEGF-B was able to do this via activating the PI-3K/ Akt-GSK-3β-mTOR signalling axis, modulating neuronal oxidative stress and increasing PEDF levels.

In a rat model of subarachnoid haemorrhage (SAH), it was determined that PEDF and 67LR expressions decreased 6 h post-induction of SAH [61]. Intranasal administration of the 34mer peptide reduced water content at the brain, pro-inflammatory cytokines, and neurological dysfunction in suffering rats. In the SAH model, PEDF-34 decreased the expression of TNF-α and IL-1β at 24 h.

Collectively, PEDF plays a critical role in nervous system specifically its neuroprotective, anti-angiogenic, and immunomodulatory functions. Beyond the eye, PEDF influences CNS cell populations, promotes nerve regeneration, and is implicated in mood disorders like depression, where reduced levels correlate with depressive-like behaviours.

## 4. PEDF and Bone

PEDF has biological roles in tissue enriched with collagen, binding to the collagen fibril, and found to be abundant in select regions of bone formation and remodelling [62]. There is a heterogeneous presence of PEDF in cortical rabbit femur, while exogenous PEDF binding becomes concentrated between highly aligned collagen fibrils. PEDF, sequestered during de novo pericellular collagen fibrils formation, become liberated as collagen crosslinking progresses, making the serpin molecules free to interact with their target cell surface receptors [63].

In a murine model of aggressive osteosarcoma, the epiphyseal cartilage was found to remain intact, despite increasing size of tumour lesion and/or intra- and extraosseous destruction [64]. Furthermore, in advanced osteosarcoma, only the regions highly expressing VEGF in the hypertrophic zone of the growth plate were impacted upon. The resting, proliferative, and upper hypertrophic layers, high in PEDF, resisted osteosarcoma invasion in all cases.

PEDF expression was demonstrated in chondrocytes within various zones of the epiphyseal growth plate [65]. PEDF was expressed by osteoblasts lining bony spicules in the ossification zone, as well as by osteoblasts on the perimeter of the cortical periosteum. The authors postulated that PEDF has a regulatory role to play in chondrocyte and osteoblast differentiation, ossification of endochondral tissue, bone modelling, and remodelling during expansion and maturation of long bones. In adult bones of mice, PEDF localised to ridges of trabecular bone in tibial cortex and to megakaryocytes within bone marrow [66]. In that study, both Hsp47 and collagen I were associated with developing mouse bone. Immunohistochemical staining in adult and foetal bone mirrors collagen I. In osteosarcoma cells, PEDF increases the expression of collagen I, HSP47, and MT1-MMP, while decreasing the expression of MMP-2.

Alginate beads incorporated with PEDF protein embedded in intramuscular pockets were found to produce de novo bone tissue, evidenced by osteoid tissue [67]. Micro-CT, histology (H&E, Alcian blue) and immunohistochemistry (ALP, OCN, OPN, collagen I) for bone markers and collagen I-processing proteins (MT1-MMP, Hsp47) confirmed osteogeneration induced by PEDF-containing beads.

Apart from muscle tissue, PEDF is capable of promoting transdifferentiation of adipocytes to osteoblasts [68]. It promotes bone formation in cultured adipocytes, as demonstrated by de novo bone formation in gelfoam fatpad implants in mice. However, mechanistic evidence is currently lacking a better understanding of this phenomenon. In this study, it was found that bone formation in white adipose tissue (WAT) was superior to that in brown adipose tissue (BAT).

In a murine study, the alveolar bone volume and density in PEDF KO animals were reduced compared to their WT counterparts [69]. Elevated receptor activator for nuclear factor-κB ligand (RANKL) expression and dampened osteoprotegerin (OPG) levels were noted in PEDF KO mice. In periodontal ligament fibroblasts, PEDF dose-dependently improved mineral deposition, promoting OPG and inhibiting RANKL, GSK3b mRNA, Wnt5a, and non-phosphorylated β-catenin protein expressions. Furthermore, as the cell culture reached seven days, RUNX2 and ALP were upregulated, whereas VEGF was downregulated post-treatment with PEDF.

Thus, to summarise the above, PEDF is involved in collagen fibril assembly as it binds to collagen fibril and there are high levels of the serpin in regions of active osteogeneration [66]. PEDF binds heterogeneously in cortical rabbit femur [62]. Osteogenesis imperfecta (OI) is a disease where several mutations in the PEDF gene have been noted, with defects in proper PEDF functioning and severe bone deformities and fracture risks [70]. Atypical collagen fibril organisation seen in perilacunar region of young osteocytes from young OI patients indicates a disturbance in early mineralisation [71].

OI patients with *SERPINF1* gene variants seem to have impaired response to anti-osteoporotic treatments. A study of OI patients in India [72] found elevated alkaline phosphatase levels in all children at their initial visit. Patients also presented with low bone mineral density, with only 7 out of 18 children showing improvement after two years of pamidronate treatment. Following denosumab treatment, iliac bone showed no change in parameters typical of OI type VI, though osteoclast numbers in trabecular bone still increased [73]. The osteoclast-suppressing effect of denosumab does not last long in children with OI type VI when compared to adults suffering from osteoporosis, plus it seems that osteoclasts bounce back after cessation of denosumab therapy.

*SERPINF1* (PEDF) is implicated in type VI osteogenesis imperfecta (OI), for which several null recessive mutation variants have been reported [74,75]. In an adult with OI in (deletion in exon 8), calcification (‘popcorn’) in both femoral epiphyses was noted [76]. One presented itself suspiciously as neoplasia, later being identified as chondrosarcoma. In another study, a homozygous variant that generates an alternative mutation in intron 4 of *SERPINF1* was identified to cause severe bone fragility in OI [77].

SERPINF1 -/- do not present with fractures until after 6 months, suggesting existence of a protective effect of maternal PEDF during foetal development, due to supposed placental passage [78], which requires further evaluation. Asymptomatic heterozygous parents have PEDF blood levels of about 1 μg/mL [79], lower than normal of ~5–10 μg/mL but sufficient for bone homeostasis as *SERPINF1* mutation carriers have no detectable abnormalities in bone and fat [80]. However, stress fractures can develop in heterozygous carriers [81].

PEDF induces mesenchymal stem cells (MSCs) to the osteoblast lineage by influencing Wnt/b-catenin signalling, though the exact molecular signalling has yet to be properly defined [82,83]. Gene expression data of *Serpinf1* in a plethora of mouse tissues revealed that PEDF is extremely highly expressed in osteoblasts (days five, fourteen, and twenty-one), a 250-fold higher than the median level across multiple tissues [84,85]. PEDF boosted volume of trabecular bone and total volume in six-month-old PEDF KO mice but not in their wildtype counterparts, and enhanced bone plasticity [86]. Heightened serum PEDF level does not improve bone phenotype by increasing osteoid and decreasing bone mass in *Serpinf1* KO mice [87].

Dexamethasone (DEX) downregulates PEDF expression, causing osteoblast death. PEDF inhibits DEX-induced cell apoptosis [88]. PEDF protein levels were higher in clinical osteoarthritic samples [89]. PEDF-deficient bones from 29-week-old mice displayed less matrix loss in response to IL-1β. In addition, PEDF-deficiency in these mice preserved matrix integrity and protected against cell loss in the joint destruction model. When mice were injected with PTH, increased PEDF expression was noted in microvascular cells, causing a decrease in angiogenesis and potential of differentiation of endothelial cells towards osteoblastic cells [90].

In *Serpinf1* KO mice and primary osteoblasts, there is delayed maturation of osteoblasts as well as extracellular matrix mineralisation [91]. Sustained Wnt3a treatment suppressed PEDF expression and resulted in impaired osteoblast maturation [86]. The authors showed that the phenomenon was rescued by co-administering with a 34-mer PEDF peptide with a single amino acid mutation during the last 8 d of differentiation protocol.

Global transcriptome analysis by RNASeq of KO mouse osteoblasts revealed angiogenesis and osteogenesis were most impacted. MSCs propagated in osteogenic medium increased VEGF and PEDF but both factors were kept in balance during osteoblastic differentiation [92]. Human MSCs exposed to PEDF activated Erk signalling, while inhibition of Erk signalling reduced VEGF levels. Thus, PEDF regulates VEGF expression in MSCs via the Erk signalling pathway. Addition of PEDF to osteoblastic cultures led to phosphorylation of glycogen synthase kinase 3-beta (GSK-3β) and Erk, and accumulation of non-phosphorylated β-catenin [93]. It was also noted that PEDF increases as VEGF increases (not quite the opposite direction like in other systems).

We discussed effects of PEDF on pre-osteoblasts and osteoblasts, but what about osteocytes? When primary osteocytes from human bone fragments were exposed to PEDF, decreased expression of Sost/Sclerostin and matrix phosphoglycoprotein as well as dentin matrix protein (DMP-1) were noted [94]. Intriguingly, PEDF reduced overall protein synthesis in osteocytes by 50%, perhaps leaning towards senescence as it does in fibroblasts, though studies dedicated to this are warranted.

## 5. PEDF and the Immune System

In PEDF null mice, upregulation of interleukin-2 (IL-2) and granulocyte–macrophage colony-stimulating factor (GM-CSF) have been noted [12]. There were also increases in white blood cells (WBCs), red blood cells (RBCs) and platelets found in serum of these mice, as well as an increase in serum C-reactive protein (CRP). PEDF enhances plaque stability by PPAR-γ-mediated anti-inflammation in macrophages [95]. PEDF reduces IL-8 production through suppression of nuclear factor kappa B (NF-κB) transactivation in prostate cancer cells [96]. PEDF can decrease the expressions of monocyte chemoattractant protein (MCP-1), vascular cell adhesion molecule-1 (VCAM-1), and plasminogen activator inhibitor-1 (PAI-1) in mesangial cells via NF-κB inactivation [97]. Thus, PEDF exerts anti-inflammatory effects partially by suppressing NF-κB.

PEDF dampened tumour necrosis factor-a (TNF-a)-mediated loss of cell viability in ARPE-19 cells and dampened IL-6 expression [50]. PEDF peptides, pro-survival 44-mer and 17-mer H105A reduced TNF-α-mediated reduction in cell viability, and halted IL-6 secretion. PEDF inhibited expression of inflammatory factor such as TNF-a, IL-6, and IL-1b, and progression of disease and reduced death of lung cells in animal model of acute lung injury (ALI) [98]. PEDF inhibited lipopolysaccharide (LPS)-evoked inflammatory damage and apoptotic death of RLE-6TN cells. PEDF inhibited epithelial cell injury by boosting PPAR-γ expression.

In humans, PEDF levels are higher in osteoarthritic samples compared to normal specimens [89]. In primary human articular chondrocytes, the serpin increased catabolic gene expression in the presence of IL-1b, causing significant cartilage matrix loss in whole bone organ cultures. PEDF -/- bones from 29-week-old animals had decreased matrix loss in response to IL-1b, with maintained matrix integrity and protection against loss of cells in the monoiodoacetate (MIA)-stimulated joint destruction murine model.

Administration of PEDF topically provided rats in a corneal transplantation model with an improved graft survival rate, reduced haemangiogenesis, and immune cell infiltration into the cornea, in particular, type 17 Th cells and Treg cells. Nerve reinnervation in the grafts was better in PEDF-treated recipient rats. Exogenous PEDF application in a herpetic simplex keratitis model altered degeneration of corneal nerves, neovascularisation, and repaired impaired corneal sensitivity [32]. While PEDF attenuated neutrophils, it had no effect on macrophage or CD4+ T-cell infiltration, reducing expressions of IL-1b, IL-6, TNF-a, and VEGF.

PEDF protein production levels by circulating endothelial progenitor cells (CEpCs) were upregulated in dry eye disease (DED) [99]. Mediated by PEDF, CEpCs from DED mice suppressed expression of CD86 and MHC-II in dendritic cells. Intriguingly, topical PEDF enhanced the suppression on DC maturation, reduced pro-inflammatory cytokine expression in the conjunctiva, and thereby reduced disease severity. Furthermore, PEDF promotes the immunosuppressive capability of regulatory T cells (Tregs) and alters their type 17 Th-mediated dysfunction in DED, thus suppressing DED [100].

TNF-a and apoptosis of decidual stromal cells (DSCs) following lipopolysaccharide (LPS) stimulation were both reduced by PEDF [101]. PEDF secreted from decidual natural killer (dNK) cells protected DSCs from LPS nuclear factor kappa-B (NF-kB) inhibition and provided protection to DSCs from LPS-mediated apoptotic death via inducing extracellular signal-regulated kinase (ERK) expression.

In a study where LPS-induced human chorionic trophoblast HTR8/SVneo cell model was used to replicate missed abortion (MA) in vitro [102], PEDF reversed the increase in cytidine monophosphate kinase 2 (CMPK2) expression and activation of the nucleotide-binding oligomerisation domain-like receptor protein 3 (NLRP3) inflammasome axis. By performing these, PEDF downregulated mitochondrial reactive oxygen species (ROSs) production and DNA release, lowered lactate dehydrogenase (LDH) release, and maintained cell viability.

In a three-week skin expansion protocol, increased PEDF expression was accompanied by dermal thinning [103]. Exogenous PEDF caused dermal thinning and increase in the presence of M1 macrophages in expanded skin. Under hypoxia, PEDF promoted macrophage polarisation to the M1 subtype.

PEDF expression is dampened in human pancreatic cancer samples in comparison to benign tissue, and patients with low levels of PEDF displayed increased inflammation and/or fibrosis in tumour tissue [104]. PEDF neutralised macrophage migration and blocked macrophage-induced proliferation of tumour cells. Furthermore, PEDF reduced pro-inflammatory/pro-fibrotic cytokine synthesis, reduced TGFβ synthesis by pancreatic stellate cells, and decreased collagen I deposition, thus dampening fibrosis.

Exosomes derived from poorly metastatic murine melanoma cells can inhibit pulmonary metastasis [105]. In bone marrow, these exosomes evoke an innate immune response through the expanding patrolling monocytes (PMo), which eradicates cancer cells via recruiting NK cells and macrophages.

PEDF induces pro-cancer macrophage migration in spheroid and 2D culture models [106]. PEDF increased cultured prostate cancer cell phagocytosis via apoptosis and stimulated the superoxide production in macrophages. In a preceding study, the authors established that PEDF stimulated the migration of monocytes and macrophages [107]. In prostatitis and malignant cancer, macrophages are increased in number. PEDF mRNA was downregulated in prostate cancer and prostatitis. PEDF correlated positively with macrophage density, and it stimulated inducible nitric oxide synthase (iNOS), IL12, and TNF-a whilst decreasing IL10 and arginase 1 in macrophages, pointing to an M1-type differentiation. Akin to this, when PEDF was expressed in MDA-MB-231 breast cancer cells and exosomes isolated, then used to treat M2 macrophages, reprogramming of M2 macrophages M2-to-M1 repolarisation, was confirmed [108].

In melanoma, the switch to malignant grade is associated with PEDF loss [109]. In melanoma, antimetastatic and immune properties of melanoma exosomes associate with presence of PEDF, whereby tumour cells deficient in PEDF expression no longer produce exosomes that incite surveillance by the immune system [105].

In summary, these data indicate the breadth of PEDF’s anti-inflammatory and immunomodulatory effects across different tissues and disease conditions, as well as how PEDF affects different types of immune cells. Intriguingly, PEDF may display anti-macrophage activity in some cancers and pro-macrophage in others. While the majority of studies point to PEDF as a promising treatment option for diseases involved with compromised immune systems, caution is needed to ensure appropriate testing is performed to rule out pro-cancerous effects that were not anticipated.

## 6. Future Directions

Apart from being a potent anti-angiogenic protein, PEDF has a variety of functions in the human body. This review has covered four of these—eye, bone, nervous system, and immune system (summarised in Table 1). For all of these, there have been attempts to use full length PEDF or its short peptides as therapeutic agents in preclinical studies. Results have been positive, but further work remains, to ensure peptides with better focussed activity are discovered, and more efficient drug delivery systems are formulated and developed for more selective and efficacious use of the protein and its peptides. Empirical testing of numerous peptides in cell culture, then testing the most promising ones in vivo in various disease models will enable finding peptides that are potentially therapeutic. Once these are found, the focus will change to drug delivery platforms to maximise peptide activity in the steps towards clinical evaluation.

## Figures and Tables

**Table 1:** Summary of studies indicating therapeutic potential of PEDF in the eye, nervous system, bone, and immune system.

Tissue	Experimental Model	Major Biological Findings	References
**Eye**	KO mouse	Macrophage infiltration, glia activation, pathologies linked to the vasculature, loss of photoreceptors, swelling of the inner plexiform layer leads to visual loss	[12]
	KO mouse	Ocular surface and lacrimal gland disturbances, myoepithelial cell death	[14]
	Cell culture	In retinal pigment epithelium (RPE) cells grown under normoxic conditions, addition of anti-VEGF agents resulted in a downregulation of PEDF	[15]
	Cell culture	Prevents apoptosis in light stressed photoreceptor cells	[19,20,21]
	Mouse	Decelerates degeneration of the retinal pigment epithelium in age-related macular degeneration	[22]
	Clinical observation	Suggested to protect the retina in rhegmatogenous retinal detachment	[24]
	Rat	Improves graft survival rate of corneal allografts	[25]
	Rat	Better than anti-VEGFs in oxygen-induced retinopathy	
**Nervous system**	Cell culture	Has pro-survival effects of PEDF on retinal ganglion cells (RGCs)	[26]
	Mouse	Improves motor behavioural performance in mouse model of lower thoracic photothrombotic ischaemia	[30]
	Mouse	Provides resistance to depression	[35]
	Mouse	Reduces apoptosis of retinal ganglionic cells (RGCs) occurring after optic nerve injury	[36]
	Cell culture	Treated RPE cells had increased glutathione levels post-H_2_O_2_ incubation	[37]
	Tissue culture	Promotes RGC survival	[39]
	Tissue culture	Promotes adult RGC neuroprotection and axon regeneration	[41]
	Mouse	Promotes significant axon regeneration with boosted physiological function in injured dorsal column	[43]
	Mouse	Ameliorates depression-like behaviours in the chronic unpredictable mild stress model	[33]
	Mice	44-mer peptide plus docosahexaenoic acid stimulates corneal nerve regeneration	[47]
	Mouse	Restores intrastrial fluid–blood barrier integrity, thereby reducing hearing loss	[51]
	Rat	Reduced outer hair cell and spiral ganglion neuron loss-mediated hearing loss	[52]
	Rat	Increases both area and number of myelinated axons	[53]
	Mouse	In an ex vivo model of ocular ischaemia/hypoxia, PEDF inhibited labyrinth angiogenesis and kept the capillary lumen patent	[54]
	Mouse	Peptides containing the neurotrophic region of PEDF decreased the number of apoptotic photoreceptors in retinal degeneration models in mice	[55]
**Bone**	Mouse	Induces mesenchymal stem cells (MSCs) to the osteoblast lineage	[82]
	Mouse	Chitosan microparticles encapsulating PEDF were shown to induce de novo bone formation in muscle pockets	[110]
	Mouse	Alginate beads incorporated with PEDF protein embedded in intramuscular pockets were found to produce do novo bone tissue	[67]
	Mouse	Promotes transdifferentiation of adipocytes to osteoblasts	[68]
	KO mouse	Boosts trabecular bone volume/total volume	[86]
**Immune system**	Mouse	Enhances the stability of atherosclerotic plaques by PPAR-γ-mediated anti-inflammation in macrophages	[92]
	ARPE-19 cells	PEDF dampened loss of cell viability	[50]
	Mouse	Inhibited expression of TNF-α, IL-6 and IL-1β, and progression of acute lung injury.	[98]
	RLE-6TN cells	Inhibits lipopolysaccharide-evoked inflammatory damage and apoptosis	[98]
	Mouse	Plays a protective role in depression	[32]
	Mouse	Enhances suppression of dendritic cell maturation, reduced pro-inflammatory cytokine expression in the conjunctiva, and thereby reduced disease severity	[100]
	Cell culture	Reduces apoptosis of decidual stromal cells (DSCs) following LPS stimulation	[101]
	Cell culture	Neutralises macrophage migration and blocks macrophage-induced proliferation of tumour cells	[104]
	Cell culture	Induces pro-cancer macrophage migration in spheroid and 2D culture models	[106]
